# Supplementary material for: Effect of Continuous Positive Airway Pressure on Lipid Profiles in Obstructive Sleep Apnea: A Meta-Analysis
Source: J Clin Med. 2022 Jan 25;11(3):596. doi: 10.3390/jcm11030596 (PMC8837151; doi:10.3390/jcm11030596)
Supplement: Supplementary file 1 [file jcm-11-00596-s001.zip › jcm-1485804-supplementary.pdf]

Supplementary Text: The unit conversion formulas of each lipid profile

Total cholesterol, high-density lipoprotein, low-density lipoprotein: 1 mmol/l=38.7 mg/dl

Triglyceride: 1 mmol/l=88.6 mg/dl

Figure S1. Funnel plots of lipid profiles

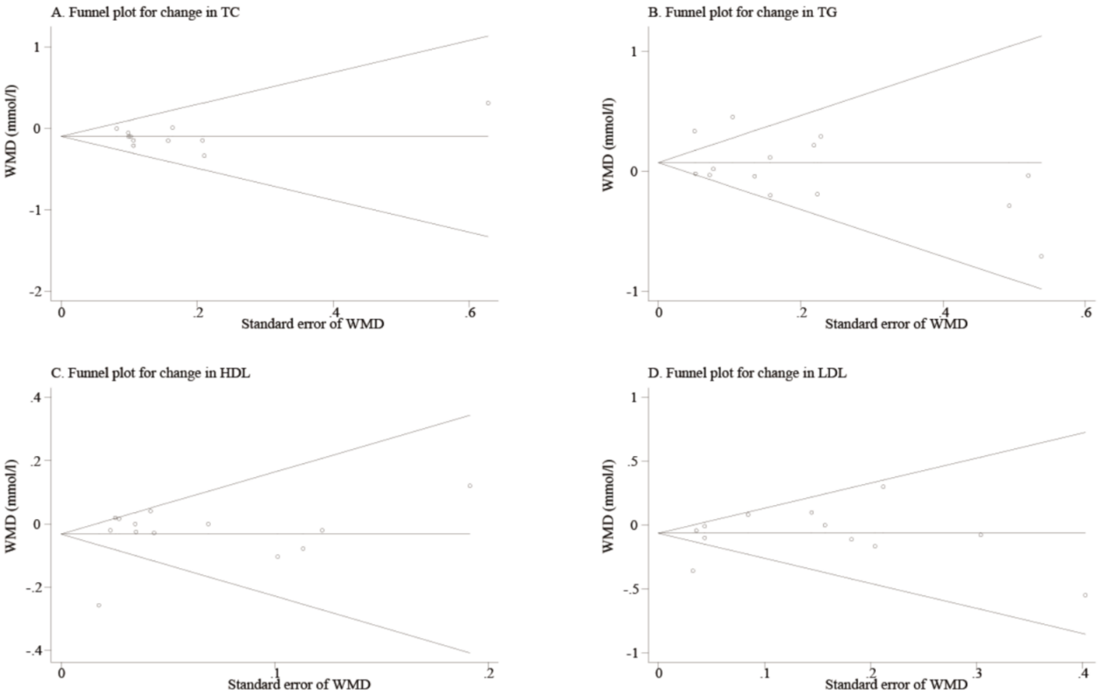

The funnel plots show no obvious asymmetry, suggesting low risk of publication bias. HDL=high-density lipoprotein, LDL=low-density lipoprotein, TC=total cholesterol, TG=triglyceride, WMD=weighted mean difference

Figure S2. Funnel plots after trim and fill for total cholesterol

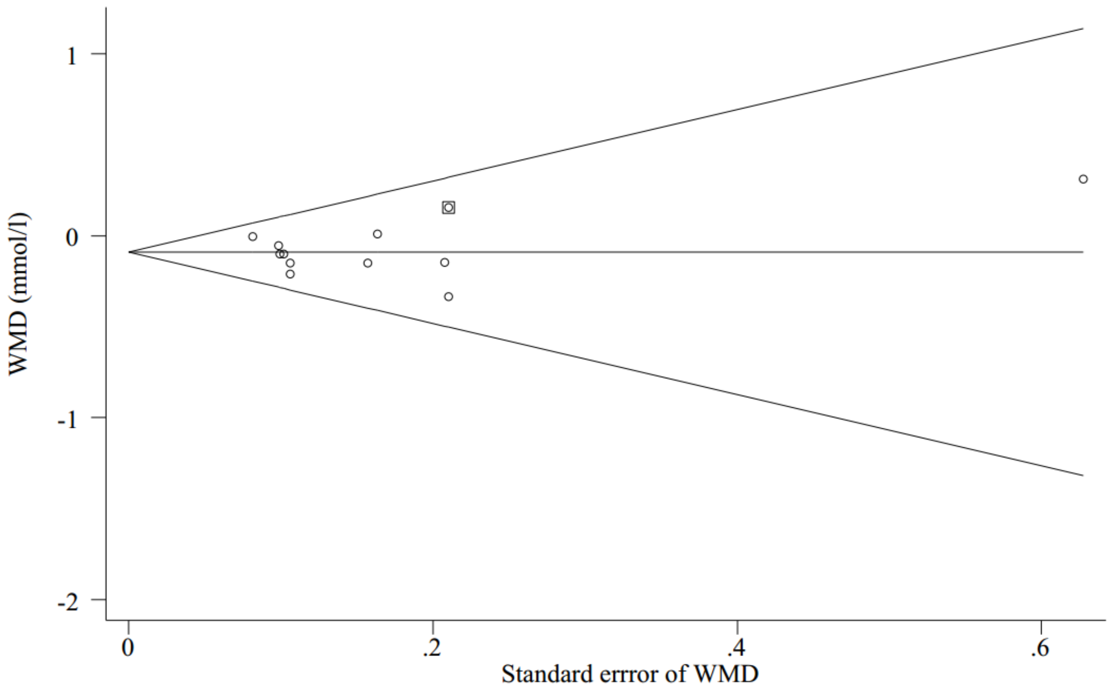

TC=total cholesterol, WMD=weighted mean difference

Table S1. Search strategies

1. Search strategies for PubMed/Medline

	Query	Hits
#1	sleep apnea, obstructive[MeSH Terms] OR sleep apnea syndrome[MeSH Terms]	38601
#2	SDB[Text Word] OR OSA[Text Word] OR OSAS[Text Word] OR SAHS[Text Word] OR SAS[Text Word] OR OSAHS[Text Word] OR obstructive sleep apnea[Text Word] OR sleep disorder breathing[Text Word] OR obstructive sleep apnoea[Text Word] OR sleep apnea syndrome[Text Word] OR sleep apnoea syndrome[Text Word]	49910
#3	#1 or #2	61715
#4	continuous positive airway pressure[MeSH Terms] OR positive pressure ventilation[MeSH Terms] OR Noninvasive Ventilation[MeSH Terms] OR positive pressure respiration[MeSH Terms]	29461
#5	PAP[Text Word] OR positive airway pressure[Text Word] OR CPAP[Text Word] OR continuous positive airway pressure[Text Word] OR NPPV[Text Word] OR positive pressure ventilation[Text Word] OR positive pressure respiration[Text Word]	56139
#6	#4 or #5	58420
#7	(randomized controlled trial [pt] OR controlled clinical trial [pt] OR randomized [tiab] OR randomised [tiab] OR placebo [tiab] OR clinical trials as topic [mesh: noexp] OR randomly [tiab] OR trial [ti]) NOT (animals [mh] NOT humans [mh])	1398222
#8	review[Publication Type] OR "meta analysis"[Publication Type]	2944182
#9	#7 not #8	1158596
#10	#3 and #6 and #9 Filters: Publication date to 2021/07/01	1393

2. Search strategies for Embase

	Query	Hits
#1	'sleep disordered breathing'/exp OR 'sdb':ti,ab,kw OR 'osa':ti,ab,kw OR 'osas':ti,ab,kw OR 'sahs':ti,ab,kw OR 'sas':ti,ab,kw OR 'osahs':ti,ab,kw OR 'obstructive sleep apnea':ti,ab,kw OR 'sleep disorder breathing':ti,ab,kw OR 'obstructive sleep apnoea':ti,ab,kw OR 'sleep apnea syndrome':ti,ab,kw OR 'sleep apnoea syndrome':ti,ab,kw	181069
#2	'positive end expiratory pressure'/exp OR 'noninvasive ventilation'/exp OR 'pap':ti,ab,kw OR 'positive airway pressure':ti,ab,kw OR 'cpap':ti,ab,kw OR 'continuous positive airway pressure':ti,ab,kw OR 'positive pressure ventilation':ti,ab,kw OR 'nppv':ti,ab,kw OR 'positive pressure respiration':ti,ab,kw	109930
#3	('randomized controlled trial'/exp OR 'randomized':ti,ab,kw OR 'placebo':ti,ab,kw OR 'randomly':ti,ab,kw OR 'randomised':ti,ab,kw) NOT 'review':it NOT 'meta analysis'/exp	1401269
#4	#1 AND #2 AND #3 AND [1-1-0001]/sd NOT [1-7-2021]/sd	2447

3. Search strategies for Cochrane Central Register

	Search	Hits
#1	MeSH descriptor: [Sleep Apnea, Obstructive] explode all trees	2092
#2	MeSH descriptor: [Sleep Apnea Syndromes] explode all trees	2795
#3	(SDB):ti,ab,kw OR (OSA):ti,ab,kw OR (OSAS):ti,ab,kw OR (SAHS):ti,ab,kw OR (SAS):ti,ab,kw	7795
#4	(OSAHS):ti,ab,kw OR (obstructive sleep apnea):ti,ab,kw OR (sleep disorder breathing):ti,ab,kw OR (obstructive sleep apnoea):ti,ab,kw OR (sleep apnea syndrome):ti,ab,kw	6553
#5	(sleep apnoea syndrome):ti,ab,kw	2116
#6	#1 or #2 or #3 or #4 or #5	10876
#7	MeSH descriptor: [Continuous Positive Airway Pressure] explode all trees	1173
#8	MeSH descriptor: [Positive-Pressure Respiration] explode all trees	2871
#9	MeSH descriptor: [Noninvasive Ventilation] explode all trees	295
#10	(PAP):ti,ab,kw OR (positive airway pressure):ti,ab,kw OR (CPAP):ti,ab,kw OR (continuous positive airway pressure):ti,ab,kw OR (NPPV):ti,ab,kw	9999
#11	(positive pressure ventilation):ti,ab,kw OR (positive pressure respiration):ti,ab,kw	5927
#12	#7 or #8 or #9 or #10 or #11	12740
#13	(randomized control trial):pt OR (controlled clinical trial):pt OR (randomized):ti,ab,kw OR (placebo):ti,ab,kw OR (randomly):ti,ab,kw	1283486
#14	#6 and #12 and #13 Custom year range: 01/01/1900 to 01/07/2021	2687

Table S2. List of 14 included studies

[1] Robinson G V, Pepperell J C T, Segal H C, et al. Circulating cardiovascular risk factors in obstructive sleep apnoea: Data from randomised controlled trials[J]. *Thorax*,2004,59(9):777-782.

[2] Drager L F, Bortolotto L A, Figueiredo A C, et al. Effects of continuous positive airway pressure on early signs of atherosclerosis in obstructive sleep apnea[J]. *Am. J. Respir. Crit. Care Med.*,2007,176(7):706-712.

[3] Nguyen P K, Katikireddy C K, Mcconnell M V, et al. Nasal continuous positive airway pressure improves myocardial perfusion reserve and endothelial-dependent vasodilation in patients with obstructive sleep apnea.[J]. *J Cardiovasc Magn Reson*,2010,12:50.

[4] Craig S E, Kohler M, Nicoll D, et al. Continuous positive airway pressure improves sleepiness but not calculated vascular risk in patients with minimally symptomatic obstructive sleep apnoea: The MOSAIC randomised controlled trial[J]. *Thorax*,2012,67(12):1090-1096.

[5] Pedrosa R P, Drager L F, De Paula L K G, et al. Effects of OSA treatment on BP in patients with resistant hypertension: A randomized trial[J]. *Chest*,2013,144(5):1487-1494.

[6] Mcmillan A, Bratton D J, Faria R, et al. A multicentre randomised controlled trial and economic evaluation of continuous positive airway pressure for the treatment of obstructive sleep apnoea syndrome in older people: PREDICT[J]. *Health Technol Assess*,2015,19(40):1-188.

[7] Feres M C, Fonseca F A H, Cintra F D, et al. An assessment of oxidized LDL in the lipid profiles of patients with obstructive sleep apnea and its association with both hypertension and dyslipidemia, and the impact of treatment with CPAP[J]. *Atherosclerosis*,2015,241(2):342-349.

[8] Salord N, Fortuna A M, Monasterio C, et al. A randomized controlled trial of continuous positive airway pressure on glucose tolerance in obese patients with obstructive sleep apnea[J]. *Sleep*,2016,39(1):35-41.

[9] Huang Z, Liu Z, Zhao Z, et al. Effects of Continuous Positive Airway Pressure

on Lipidaemia and High-sensitivity C-reactive Protein Levels in Non-obese Patients with Coronary Artery Disease and Obstructive Sleep Apnoea[J]. *Heart Lung Circul.*,2016,25(6):576-583.

[10] Martinez-Ceron E, Barquiel B, Bezos A M, et al. Effect of Continuous Positive Airway Pressure on Glycemic Control in Patients with Obstructive Sleep Apnea and Type 2 Diabetes. A Randomized Clinical Trial[J]. *Am J Respir Crit Care Med*,2016,194(4):476-485.

[11] Lam J C M, Lai A Y K, Tam T C C, et al. CPAP therapy for patients with sleep apnea and type 2 diabetes mellitus improves control of blood pressure[J]. *Sleep* Breathing,2017,21(2):377-386.

[12] Campos-Rodriguez F, Gonzalez-Martinez M, Sanchez-Armengol A, et al. Effect of continuous positive airway pressure on blood pressure and metabolic profile in women with sleep apnoea[J]. *Eur. Respir. J.*,2017,50(2).

[13] E Silva LO, Guimarães TM, Pontes G, Coelho G, Badke L, Fabbro CD, Tufik S, Bittencourt L, Togeiro S. The effects of continuous positive airway pressure and mandibular advancement therapy on metabolic outcomes of patients with mild obstructive sleep apnea: a randomized controlled study. *Sleep Breath*. 2021 Jun;25(2):797-805. doi: 10.1007/s11325-020-02183-1. Epub 2021 Jan 4.

[14] Celik, Y.; Balcan, B.; Peker, Y. CPAP Intervention as an Add-On Treatment to Lipid-Lowering Medication in Coronary Artery Disease Patients with Obstructive Sleep Apnea in the RICCADSA Trial. *J. Clin. Med.* 2022;11(1):273. Published 2022 Jan 5. doi:10.3390/jcm11010273

Table S3. Quality and risk of bias

	Study (first author, year)	Random sequence generation	Allocation concealment	Blinding	Incomplete outcome data	Selective reporting
[1]	Robinson, 2004	Unclear	Unclear	Low	Unclear	Low
[2]	Drager, 2007	Low	Unclear	Low	Low	Low
[3]	Nguyen, 2010	Unclear	Unclear	Low	Low	Unclear
[4]	Craig, 2012	Low	Low	Low	Low	Low
[5]	Pedrosa,2013	Low	Unclear	Low	Low	Low
[6]	McMillan, 2015	Low	Low	Low	Low	Low
[7]	Feres, 2015	Unclear	Unclear	Low	High	Unclear
[8]	Salord, 2016	Low	Low	Low	High	Low
[9]	Huang, 2016	Low	Low	Low	High	Low
[10]	Lam, 2017	Low	Low	Low	Low	Low
[11]	Rodriguez, 2017	Low	Low	Low	Low	Low
[12]	Pascual, 2018	Low	Low	Low	Low	Low
[13]	Silva, 2020	Unclear	Unclear	Unclear	Low	Unclear
[14]	Celik, 2022	Unclear	Unclear	Low	Low	Low

The quality and risk of bias of studies were assessed by using Cochrane Collaboration tool.

Table S4 Comparison of total cholesterol before and after trim and fill

	Before trim and fill			After trim and fill		
	n	*WMD* (*95% CI*)	*P*	*n*	*WMD* (*95% CI*)	*P*
TC, mmol/l	11	-0.098 (-0.169 to -0.027)	0.007	12	-0.091 (-0.161 to -0.020)	0.012

TC=total cholesterol, WMD=weighted mean difference

Table S5-1. Sensitivity analyses of total cholesterol

Study omitted	Estimate	[95% Conf.	Interval]
Robinson, 2004	-0.08301325	-0.15886132	-0.00716517
Drager, 2007	-0.09626774	-0.16866001	-0.02387546
Craig, 2012	-0.09754509	-0.17414299	-0.02094721
Pedrosa, 2013	-0.09924889	-0.17064057	-0.02785721
McMillan, 2015	-0.09756524	-0.17384826	-0.02128222
Feres, 2015	-0.09106418	-0.16694887	-0.01517950
Huang, 2016	-0.10347956	-0.17657942	-0.03037971
Lam, 2017	-0.09491885	-0.16818346	-0.02165425
Rodriguez, 2017	-0.12129453	-0.20097360	-0.04161546
Pascual, 2018	-0.09054761	-0.16290791	-0.01818730
Celik, 2022	-0.10449253	-0.18120848	-0.02777657
Combined	-0.09787463	-0.16914636	-0.02660289

Table S5-2. Sensitivity analyses of triglyceride

Study omitted	Estimate	[95% Conf.	Interval]
Robinson, 2004	0.08919748	-0.04598834	0.22438329
Drager, 2007	0.06519557	-0.07219718	0.20258832
Nguyen, 2010	0.07556043	-0.05831041	0.20943126
Craig, 2012	0.08549876	-0.05914781	0.23014532
Pedrosa, 2013	0.06170956	-0.07491008	0.19832920
McMillan, 2015	0.07826278	-0.06771462	0.22424018
Feres, 2015	0.04242621	-0.07081598	0.15566841
Salord, 2016	0.03595541	-0.09342412	0.16533493
Huang, 2016	0.08407970	-0.05584837	0.22400777
Lam, 2017	0.09736572	-0.03761051	0.23234196
Rodriguez, 2017	0.08407024	-0.06234865	0.23048912
Pascual, 2018	0.08005958	-0.05325329	0.21337244
Silva, 2020	0.08562919	-0.04548026	0.21673864
Celik, 2022	0.06952607	-0.07072233	0.20977446
Combined	0.07430991	-0.05731284	0.20593266

Table S5-3. Sensitivity analyses of high-density lipoprotein

Study omitted	Estimate	[95% Conf.	Interval]
Drager, 2007	-0.03215907	-0.11349689	0.04917875
Nguyen, 2010	-0.02932063	-0.10764298	0.04900172
Craig, 2012	-0.03273138	-0.11848632	0.05302357
Pedrosa, 2013	-0.02743054	-0.10603452	0.05117344
McMillan, 2015	-0.03710326	-0.11910234	0.04489582
Feres, 2015	-0.00118933	-0.02302800	0.02064935
Salord, 2016	-0.03445792	-0.11395063	0.04503480
Huang, 2016	-0.03642977	-0.11331961	0.04046007
Lam, 2017	-0.03493189	-0.11676038	0.04689660
Rodriguez, 2017	-0.03239853	-0.11484747	0.05005040
Pascual, 2018	-0.03894426	-0.11882939	0.04094087
Silva, 2020	-0.03248797	-0.11056865	0.04559271
Celik, 2022	-0.03671269	-0.11880156	0.04537619
Combined	-0.03192984	-0.10760501	0.04374534

Table S5-4. Sensitivity analyses of low-density lipoprotein

Study omitted	Estimate	[95% Conf.	Interval]
Drager, 2007	-0.06090186	-0.18706910	0.06526539
Nguyen, 2010	-0.08444256	-0.20673917	0.03785406
Craig, 2012	-0.07114834	-0.20602511	0.06372842
Pedrosa, 2013	-0.06375055	-0.18742712	0.05992602
McMillan, 2015	-0.05738017	-0.19966134	0.08490100
Feres, 2015	-0.03700811	-0.07988618	0.00586996
Huang, 2016	-0.07829013	-0.20403281	0.04745254
Lam, 2017	-0.06920213	-0.19578452	0.05738028
Rodriguez, 2017	-0.08224992	-0.20926459	0.04476476
Pascual, 2018	-0.05821997	-0.18372943	0.06728949
Silva, 2020	-0.05445078	-0.17658950	0.06768794
Celik, 2022	-0.06550074	-0.20676067	0.07575919
Combined	-0.06434616	-0.18514938	0.05645705

Table S6. Meta-regression analyses for lipid profiles

Independent variable	Outcome (*coefficient*, *P*-value）			
	ΔTC	ΔTG	ΔHDL	ΔLDL
Age, year	0.006, 0.339	-0.009, 0.347	0.003, 0.439	0.004, 0.567
Male percentage, %	-0.002, 0.186	-0.001, 0.441	0.000, 0.445	-0.001, 0.205
BMI, kg/m^2	-0.008, 0.503	0.016, 0.146	0.002, 0.712	-0.005, 0.810
Obesity	-0.055, 0.543	0.059, 0.708	-0.034, 0.594	-0.100, 0.408
ESS, point	-0.013, 0.319	-0.015, 0.576	-0.001, 0.854	-0.006, 0.568
AHI, event/hour	-0.003, 0.328	0.008, 0.057	0.000, 0.970	-0.003, 0.549
Follow-up duration, week	0.002, 0.493	-0.002, 0.728	0.000, 0.710	-0.002, 0.672
CPAP compliance, hour/night	-0.003, 0.921	0.067, 0.135	-0.005, 0.567	0.032, 0.199
Cardiometabolic disease	0.052, 0.553	-0.079, 0.594	0.041, 0.542	0.093, 0.451

AHI=apnea-hypopnea index; CPAP=continuous positive airway pressure; ESS=Epworth Sleepiness Scale; HDL=high-density lipoprotein, LDL=low-density lipoprotein, TC=total cholesterol, TG=triglyceride, WMD=weighted mean difference

We meta-regressed one variable at a time. Each Δlipid profile was calculated as the endpoint minus baseline values.

Cardiometabolic disease was defined as resistant hypertension, coronary artery disease, or diabetes. Obesity was defined based on the studies in which mean BMI ≥30 kg/m^2.
